# Delirium in elderly inpatients admitted to clinical wards Prevalence and investigation of clinical conditions in a Brazilian sample

**DOI:** 10.1590/1980-57642018dn12-020007

**Published:** 2018

**Authors:** Fernando de Bortoli Pereira, Marcos Antonio Lopes

**Affiliations:** 1 Health Science Center, Internal Medicine Department, Federal University of Santa Catarina, Florianópolis, SC, Brazil.

**Keywords:** delirium, aged, hospitalization, consultation, epidemiology, delirium, idoso, hospitalização, interconsulta, epidemiologia

## Abstract

In Brazil there is scarce data about the occurrence of *delirium* among hospitalized elderly patients.

**Objective:**

This study aimed to evaluate the prevalence of *delirium* among elderly patients hospitalized in clinical wards.

**Methods:**

This cross-sectional study examined a sample of elderly inpatients admitted to three clinical wards of a general hospital between July 2011 and May 2012. The presence of delirium was detected by applying the Confusion Assessment Method (CAM). Dementia diagnosis was conducted in two steps: screening and diagnosis (Cambridge Examination, CAMDEX, was applied during hospitalization at a second timepoint). Other medical diagnoses and medications in use were extracted from medical records.

**Results:**

A sample of 173 elderly inpatients was examined; mean age 71.2 years (SD: 7.8; 60-92 years); 64.2% male. Thirty-one patients were diagnosed with *delirium;* prevalence of 17.9% (95% CI: 12.2-23.6). Delirium was directly associated with Urinary Tract Infection, Renal Failure and Dementia (p<0.05).

**Conclusion:**

The principal findings of this study were a high prevalence of *delirium* and the identification of associated factors, helping to guide preventive approaches and clinical management for at-risk patients in a Brazilian sample.

## Resumo

No Brasil há poucos dados sobre a ocorrência de *delirium* entre pacientes idosos hospitalizados.

**Objetivo:**

Este estudo teve o objetivo de avaliar a prevalência de *delirium* entre pacientes idosos hospitalizados em enfermarias clínicas.

**Métodos:**

Este estudo transversal examinou uma amostra de idosos hospitalizados em enfermarias clínicas de um hospital geral, no período de julho de 2011 a maio de 2012. A identificação de *delirium* foi feita através da aplicação do *Confusion Assessment Method* (CAM). O diagnóstico de demência foi conduzido em duas etapas: rastreamento e diagnóstico (o *Cambridge Examination*, CAMDEX, foi aplicado durante a hospitalização em um segundo momento). Os outros diagnósticos médicos e as medicações em uso foram extraídos dos prontuários médicos.

**Resultados:**

Uma amostra de 173 idosos internados foi examinada; média de idade de 71,2 anos (DP: 7.8; 60-92); 64,2% eram homens. Trinta e um pacientes foram diagnosticados com *delirium;* prevalência de 17,9% (IC 95%: 12,2-23,6). *Delirium* exibiu uma associação direta com infecção urinária, insuficiência renal e demência. (p<0,05).

**Conclusão:**

A principal implicação deste estudo foi prover informação útil - alta prevalência de delirium e identificação de fatores associados - com o propósito de orientar abordagens preventivas e manejo clínico dirigidos a pacientes com maior risco em uma amostra brasileira.

According to a well-established definition, *delirium* is a neurobehavioral syndrome caused by the acute and transient impairment of brain activity. The clinical context usually involves a vulnerable patient, susceptible to predisposing factors, such as clinical illness, cognitive and sensory deficits, with precipitating factors occurring during the period of hospitalization.^1^ It is highly prevalent in clinical wards, being considered the most common complication of hospital admission in elderly people.^2^ Although this medical emergency is cited as one of the oldest diseases described in medicine, its pathophysiological mechanisms are not well defined.

Studies estimate failure to recognize *delirium* by physicians in up to 70% of cases,^3^ a cause of concern since failure to detect the disease in clinical emergencies is associated with a 7-fold increase in mortality.^4^ This disease is directly associated with a worse prognosis,^5^ being related, among other factors, to a 2-fold increase in mortality, an average of eight additional days of hospital stay, worsening of physical and cognitive recovery after 1 year of hospitalization and longer institutionalization time after discharge.^6^ A systematic review analyzing the occurrence of delirium and its consequences in hospitalized patients evaluated 42 studies and found rates ranging from 11% to 42%.^7^ Regarding Brazilian data, an article analyzing the prevalence and incidence of delirium among elderly hospitalized with hip fractures revealed rates of 16.5% and 12.6%, respectively.^8^

Although widely explored in international studies, there is little knowledge about the occurrence of *delirium* among hospitalized patients in Brazil, especially among elderly patients, the population most affected by this clinical condition. The importance of the present article, therefore, lies in filling this gap in the national literature, focusing on a condition of extreme relevance in the clinical context of general hospitals in Brazil. The objectives of this article were to evaluate the prevalence of delirium among elderly patients hospitalized in the clinical wards of the University Hospital of the Federal University of Santa Catarina (HU-UFSC) and its distribution in relation to sociodemographic characteristics, clinical conditions and medications.

## METHODS

### Study design and sample

This cross-sectional study was conducted at the HU-UFSC in Florianópolis (approximately 400,000 population, of which 10.8% were aged 60 years and older, according to the 2010 census). In the study period, 55% of the 77 clinical ward beds at the HU-UFSC were occupied by elderly people. All elderly patients (60 years of age or older) admitted to the Medical Clinic Units of the HU-UFSC between July 2011 and May 2012 for a hospital stay of up to 30 days were included in the sample. The macro-region of Florianópolis had ﬁve general hospitals. The principal causes of admission of elderly people to general hospitals in this region were related to cardio-circulatory (30.1%), oncologic (16.9%), respiratory (14.1%), and digestive (10.0%) diseases.

### Instruments

The Confusion Assessment Method (CAM) instrument was used for detecting *delirium*. The CAM is an instrument created for the diagnostic evaluation of *delirium*,^9^ with sensitivity of 94.1%, specificity of 96.3% and inter-rater reliability of 0.70.^10^ The diagnosis of *delirium* requires the presence of items 1 and 2 of the CAM (acute onset and attention disorder) and/or items 3 and 4 (disorganized thinking and altered level of consciousness). This instrument is associated with satisfactory validity and reliability, and is greater when involving multiple clinical observations.^11^

A questionnaire collecting socioeconomic and medical data was applied to the informant/relative, obtaining data regarding age, sex, marital status, education, cognitive and affective symptoms, as well as the patient’s medical history. The medical diagnoses and medications in use by patients during the hospital stay were extracted from medical records.

An investigation for the diagnosis of dementia was also performed, in two steps: screening and diagnosis. In the screening phase, the Mini-Mental State Examination (MMSE)^12^ and the Bayer-Activities of Daily Living scale (B-ADL)^13^^,^ 14 for the six months prior period were used. Similarly to a study that investigated dementia in patients with *delirium* using a scale for informants,^15^ the present study applied the B-ADL scale to the informant/relative to screen dementia. The B-ADL scale has been previously tested for differentiating between mild-to-moderate dementia and normal aging, with sensitivity and specificity of 87.9% and 96.6%, respectively.^16^ At a second timepoint during hospitalization, in the diagnostic phase, the Cambridge Examination (CAMDEX)^17^ was partially applied to the positively screened cases and informant/relative. Subsequently, a clinical discussion with a geriatric psychiatrist was conducted and the diagnosis established based on the criteria established by the Diagnostic and Statistical Manual of Mental Disorders, 4th edition (DSM-IV).^18^ The findings of the dementia investigation have been published elsewhere.^19^

### Statistical analysis

The data were analyzed using SPSS version 18.0 for Windows. Proportions, means and standard deviations (SD) were calculated. Bivariate analysis was used to compare the frequency of controls and cases of *delirium* (dependent variable) in relation to several independent variables (sociodemographic factors, clinical conditions and use of medications), employing the Chi-square test. Odds Ratios (OR) and a 95% Confidence Interval (95% CI) were calculated; a P-value <0.05 was considered statistically significant. The multivariate analysis, employing the logistic regression method, was conducted to examine the interference of the independent variables in the association with the dependent variable.

### Ethics statement

The project was approved by the local research ethics committee (CEPSH-UFSC). All participants (elderly or relatives) were required to sign a consent form.

## RESULTS

During the study period, 296 elderly persons were admitted, of which 123 (41.5%) were subject to attrition, leading to a final sample of 173 subjects. The principal reasons for attrition included medical discharge before the interview (64.6%), death (16.1%), long hospital stay (7.6%) and voluntary drop-out from the study by relatives (7.4%). The sample comprised predominantly subjects who were male (64.2%), had a low level of education (≤4 years, 73.5%) and were married (60.1%); with a mean age of 71.2 years (SD: 7.8; 60-92 years) (Table 1). A total of 31 patients (17.9%; 95% CI: 12.2-23.6) were diagnosed with *delirium*. The association of *delirium* with sociodemographic factors, several clinical conditions and use of medications was determined. The bivariate analysis revealed no association with the sociodemographic variables. Regarding clinical variables, presence of *delirium* was directly associated with urinary tract infection (UTI), renal failure, cardiac failure and dementia. With respect to medications, use of morphine was directly associated with presence of *delirium* (Table 2). Age, gender, education, marital status, chronic obstructive pulmonary disease, pneumonia, stroke, hypertension, diabetes mellitus, cancer, angiotensin-converting enzyme inhibitor, losartan, atenolol, digoxin, hydrochlorothiazide, simvastatin, metoclopramide, ranitidine, omeprazole, tramadol, dipyrone, paracetamol, acetylsalicylic acid, prednisone, hydrocortisone, insulin, heparin, marevan, ciprofloxacin, levofloxacin, amoxicillin with clavulanate, spirolactone, furosemide, hydralazine and benzodiazepine had no association with the presence of *delirium*.

**Table 1:** Sociodemographic distribution of sample.

		N	%
**Age (range)**	60-69	81	46.8
	70-79	61	35.3
	≥80	31	17.9
**Gender**	Male	111	64.2
	Female	62	35.8
**Education (years)**	0 (illiterate)	24	14.1
	1-4	101	59.4
	5-8	24	14.1
	≥9	21	12.4
**Marital status**	Single	7	4.0
	Married	104	60.1
	Divorced	11	6.4
	Widower/widow	51	29.5
Day of evaluation mean (SD; minimum-maximum)	8.7 (5.9; 1–28)		

**Table 2:** Comparison of participants with and without delirium for sociodemographic variables, clinical conditions and medications.

		Without delirium			With delirium		p**	OR	95%CI
		N	%		N	%			
Age (range)	60-69	70	86.4		11	13.6	0.162		
	70-79	50	82		11	18			
	≥80	22	71		9	29			
UTI*	No	130	85		23	15	0.018	3.29	1.17-9.25
	Yes	12	63.2		7	36.8			
Renal failure	No	129	86		21	14	0.002	4.25	1.61-11.18
	Yes	13	59.1		9	40.9			
Cardiac failure	No	135	84.4		25	15.6	0.022	3.85	1.13-13.12
	Yes	7	58.3		5	41.7			
Dementia	No	97	91.5		9	8.5	<0.001	6.46	2.21-18.89
	Yes	15	62.5		9	37.5			
Morphine	No	137	84		26	16	0.006	5.26	1.42-19.50
	Yes	5	50		5	50			

* UTI: urinary tract infection;

** Chi-square test.

On the multivariate analysis, the presence of UTI, renal failure and dementia were directly associated with *delirium* (Table 3).

**Table 3:** Multivariate analysis with clinical conditions and medications

	OR	95%CI	p**
UTI*	4.69	1.13-19.52	0.033
Renal failure	6.52	1.65-25.80	0.007
Cardiac failure	2.16	0.26-17.60	0.470
Dementia	5.24	1.45-18.85	0.011
Morphine	4.25	0.63-28.58	0.136

* UTI: urinary tract infection;

** logistic regression method

## DISCUSSION

The principal findings of this study were a high prevalence of *delirium* in the elderly inpatients and a direct association of *delirium* with UTI, renal failure and dementia.

The present study confirmed the high prevalence of *delirium* among elderly inpatients. This rate was very similar to that reported in Brazilian elderly patients admitted to a geriatric orthopedic ward,^8^ but lower than the prevalence of up to 38.3% found in world studies evaluating a geriatric sample.^20^ The considerable disparity compared with previous studies is probably related to differences among the populations evaluated, such as patients in the intensive care unit, clinical or surgical wards and inconsistency of diagnostic criteria.^20^ The clinical ward setting and the specific scale in this study might explain the relatively low prevalence detected. Moreover, the relatively low rate observed in the other Brazilian study,^8^ although conducted in an orthopedic ward, was probably due to use of two procedures within the same study, the prevalence at admission and the incidence during the hospital stay. Some studies, however, have associated the prevalence of *delirium* with the quality of the hospital service,^2^ lending greater statistical importance to this data.

The association of *delirium* with certain clinical conditions also corroborated previous studies which observed a strong association with dementia^21^ and identified infectious conditions and metabolic disorder among the main causes of *delirium* in elderly inpatients.^22^ Between 25% and 75% of patients with *delirium* have dementia, and the presence of dementia increases the risk of developing delirium by five.^21^ A study evaluating the clinical profile of elderly inpatients admitted with UTI found that *delirium* was the most common clinical manifestation, occurring in 56.3% of these patients.^23^

In relation to the present findings involving morphine on the bivariate analysis, it is important to note that, among the precipitating factors of *delirium*, medications are implicated in up to 40% of cases^1^ and represent an isolated factor in 12%-39%.^24^ Moreover, the incidence of *delirium* increases in direct proportion to the number of medications used.^1^ The medications most commonly associated with *delirium* are psychoactive medications (such as benzodiazepines), analgesics (such as morphine) and drugs with an anticholinergic effect.^24^

The main limitations of the present study were the attrition rate (41.5% of initial sample) and the small sample size, which may have led to inaccuracy in the findings (e.g. wide confidence interval on bivariate and multivariate analyses). In addition, although a globally accepted and recognized scale was used for the identification of *delirium*, another limitation was the non-inclusion of a medical evaluation to confirm the diagnosis of *delirium*.

In conclusion, the findings of this study have significant implications for clinical practice in identifying the principal factors associated with *delirium* among hospitalized elderly in a Brazilian sample. The major contribution of this study is to provide useful information to help guide a preventive approach and clinical management for at-risk patients, allowing a reduction in delirium incidence, rapid identification and improvement in prognosis.^25^
